# Effect of Pap smears on the long-term survival of cervical cancer patients: a nationwide population-based cohort study in Korea

**DOI:** 10.4178/epih.e2022072

**Published:** 2022-09-07

**Authors:** Xuan Quy Luu, Kyeongmin Lee, Jae Kwan Jun, Mina Suh, kyu-won Jung, Myong Cheol Lim, Kui Son Choi

**Affiliations:** 1 Department of Cancer Control and Population Health, Graduate School of Cancer Science and Policy, National Cancer Center, Goyang, Korea; 2 National Cancer Control Institute, National Cancer Center, Goyang, Korea; 3 Division of Tumor Immunology, Center for Gynecologic Cancer, Research Institute and Hospital, National Cancer Center, Goyang, Korea

**Keywords:** Cervical cancer, Papanicolaou test, Mass screening, Survival

## Abstract

**OBJECTIVES:**

This study aimed to investigate the effect of cervical cancer screening by Papanicolaou (Pap) smears on the long-term survival of cervical cancer patients.

**METHODS:**

We constructed a retrospective cohort of 14,903 women diagnosed with invasive cancer or carcinoma in situ in 2008 and 2009 and followed up until December 31, 2019, by using individual-level data from 3 national databases of the Korean National Cancer Screening Program, the Korean Central Cancer Registry, and death certificates. Cox proportional-hazards regression was used to investigate the effect of cervical cancer screening on mortality.

**RESULTS:**

In total, 12,987 out of 14,867 patients (87.4%) were alive at the end of the follow-up period (median: 10.5 years). Screened patients had a 38% lower risk of cervical cancer death than never-screened patients (hazard ratio [HR], 0.62; 95% confidence interval [CI], 0.54 to 0.70). Screening was associated with 59% and 35% lower risks of death, respectively, in screened patients with localized and regional stages. Furthermore, lower HRs among women who received screening were observed in all age groups, especially women aged 50–59 years (HR, 0.54; 95% CI, 0.42 to 0.69). The lowest HR for cervical cancer death was reported among patients screened within the past 2 years (HR, 0.54; 95% CI, 0.47 to 0.63), and the HRs increased with increasing time intervals.

**CONCLUSIONS:**

Pap smear screening significantly reduced the risk of cervical cancer-specific death in Korean women across all cancer stages.

## INTRODUCTION

Cervical cancer is among the most common neoplasms in women. In contrast with the decreasing trend in the cervical cancer burden in developed countries, Global Cancer Statistics estimated 604,127 newly diagnosed cervical cancer cases worldwide in 2020, ranking it fourth in terms of new cancer cases in women [1]. Cervical cancer screening can reduce the disease burden by detecting cancer at an early stage [2], and the Papanicolaou (Pap) smear is widely accepted and recommended for screening purposes [2-9].

Even though the effectiveness of Pap smears has been addressed in several studies and reports by national cancer screening programs [2,4,5,10-15], the recommended screening interval varies among guidelines [2,4,6-9,16]. Therefore, the optimal screening interval, particularly for specific regions, remains to be determined.

Since 1999, the Korean National Cancer Screening Program (KNCSP) has provided cervical cancer screening by the Pap smear test for Korean women aged 30 years or older, with the eligibility age reduced to 20 years in 2016 [16]. Since then, the age-standardized cervical cancer incidence has decreased drastically, from 16.4 cases per 100,000 women in 1999 to 8.7 cases per 100,000 women in 2017 [17]. The annual percentage change (APC) of cervical cancer incidence was -4.6% and -2.8% for the period of 1999-2007 and 2007-2017, respectively. The decreasing trend of cervical cancer-specific mortality was further evident from the APC of -4.8% during 2003-2017 [17]. However, the trends in incidence and mortality cannot be directly used to evaluate the effectiveness of the screening program.

In 2018, the World Health Organization (WHO) announced a global call for action to eliminate cervical cancer, and the global strategy for cervical cancer elimination was adopted in 2020 with the so-called 90-70-90 targets [18]. Cervical cancer screening of 70% of women using a high-performance test is 1 of the 3 key pillars of this initiative. To reach these targets by 2030, the WHO has urged each country to adopt a high-quality screening program. For countries including Korea, where a screening program has already been implemented, there is a need to re-evaluate the current policy to improve it to the highest standard. Further, previous national studies mainly focused on the impact of cervical cancer screening on cervical cancer incidence or an intermediate outcome (e.g., the stage at diagnosis) [11,12]. However, survival—particularly the long-term survival of cervical cancer patients—has never been evaluated in the KNCSP.

Therefore, using data from the KNCSP, we conducted this study to investigate the effect of cervical cancer screening by Pap smears on the long-term survival of cervical cancer patients. The secondary objective was to evaluate the impact of screening frequency and the time interval since the last screening.

## MATERIALS AND METHODS

### Study materials

We constructed a retrospective cohort study based on individual-level data from 3 national databases. By using patients’ specific 13-digit registration numbers, we linked the KNCSP database to the Korean Central Cancer Registry (KCCR) database and the Causes of Death Statistics from Statistics Korea, covering over 95% and 99% of new cases of cancer and deaths in Korea [17], respectively.

The baseline population of our study included 14,903 women aged 30-79 years who were recorded with invasive cancer or carcinoma in situ (CIS) of the cervix uteri as the primary cancer in the KCCR between January 1, 2008, and December 31, 2009, and invited to the KNCSP for cervical cancer screening. We further excluded 36 patients who died from cervical cancer before being registered in the KCCR (referred to as “death certificate only” cases). As a result, our final analysis included 14,867 patients.

### Study outcome

The major outcome of our study was the long-term survival of CIS or invasive cervical cancer patients. The date and cause of death were retrieved from the death statistics from Statistics Korea through 2019, which enabled us to follow all cervical cancer patients for at least 10 years. The main focus of our study was cervical cancer-specific death, which was identified by utilizing the International Classification of Diseases, 10th revision (ICD-10) codes C53 and D06 [19]. Additionally, all deaths, including and excluding cervical cancer-related deaths were also evaluated to account for methodological biases, such as misclassification and competing risks. The person-time of the study population was measured from the date of cancer diagnosis to that of death or the final follow-up (December 31, 2019), whichever came first.

### Measurements

We obtained information on the diagnosis of cervical cancer, date of diagnosis, tumor behavior, and characteristics from the KCCR database. First, cervical cancer patients were identified by the ICD-10 code of C53 for invasive cancer and D06 for CIS cases. Next, the stage at diagnosis was classified as localized, regionalized, distant, or unknown according to the Surveillance, Epidemiology, and End Results (SEER) summary staging system [20]. Based on the morphology codes of the International Classification of Diseases for Oncology - third edition (ICD-O-3) [21], the subtype of histology of cervical cancer patients was classified as squamous cell carcinoma, adenocarcinoma (glandular), and other tumors and precursors, following the WHO histological classification of uterine cervix tumors [22].

The study population’s screening history and socio-demographic characteristics were obtained from the database of the KNCSP. Pap smear screening history, including the test dates and results, was collected from 2002 until the cancer diagnosis date (2008-2009). Patients were labeled as never-screened and screened for the main assessment of screening history. Furthermore, the frequency and duration of screening from the previous screening round to the diagnosis of cancer were also assessed to evaluate the dose-response relationship and interval time of screening.

Based on the age at diagnosis, patients were categorized into the following age groups: 30-39 years, 40-49 years, 50-59 years, 60-69 years, and 70-79 years. In this study, we used the premium status of health insurance policies as an approximate measurement of socioeconomic status, according to which, the participants in the study were divided into 3 groups: recipients of the Medical Aid program (people who are poor and rely on government assistance for living expenses), National Health Insurance Service (NHIS) beneficiaries with a 50% or lower premium, and NHIS beneficiaries with a premium over 50%.

### Statistical analysis

The baseline characteristics and long-term survival of the study cohort were presented using descriptive statistics according to patients’ screening history. The chi-square test was used to compare the screened and never-screened patients. Kaplan–Meier analysis with the log-rank test was conducted to illustrate and compare survival between the screening history subgroups.

We used Cox proportional-hazards regression analysis to evaluate the impact of Pap smear screening on cervical cancer deaths by reporting the hazard ratios (HRs) with 95% confidence intervals (CIs). The models were initially adjusted by age and socioeconomic standing and then adjusted by cancer site, histological subtype, and cancer stage. All-cause mortality, including and excluding cervical cancer-related deaths, was also assessed to adjust for possible methodological biases. Patients who died from any cause except cervical cancer, and cervical cancer, were censored in models for cervical cancer deaths and all-cause deaths except cervical cancer, respectively. Additionally, we conducted subgroup analyses by age and cancer stage to assess variation in the effect of screening on mortality between subgroups. All statistical analyses were conducted using SAS version 9.4 (SAS Institute Inc., Cary, NC, USA), and p-values < 0.05 were considered to indicate statistical significance.

### Ethics statement

The current study was approved by the Institutional Review Board of the National Cancer Center, Korea (No. NCCNCS08129). The written consent form was obtained from the screeners in the KNCSP database for the collection of screening results. Owing to the use of de-identified data, the requirement for an informed consent form was waived for this study.

## RESULTS

The baseline characteristics of 14,867 cervical cancer patients according to their screening history are listed in Table 1. Overall, a large proportion of patients aged 40-49 at cancer diagnosis (38.5%) had an NHIS premium of lower than 50% (54.4%), a diagnosis of CIS (58.6%), and the histological subtype of squamous cell carcinoma (81.7%). Among all cervical cancer patients, 8,418 (56.6%) were screened for cervical cancer by a Pap smear at least once. The distribution of all baseline characteristics was significantly different between the never-screened and screened patients.

**Figure 1.**
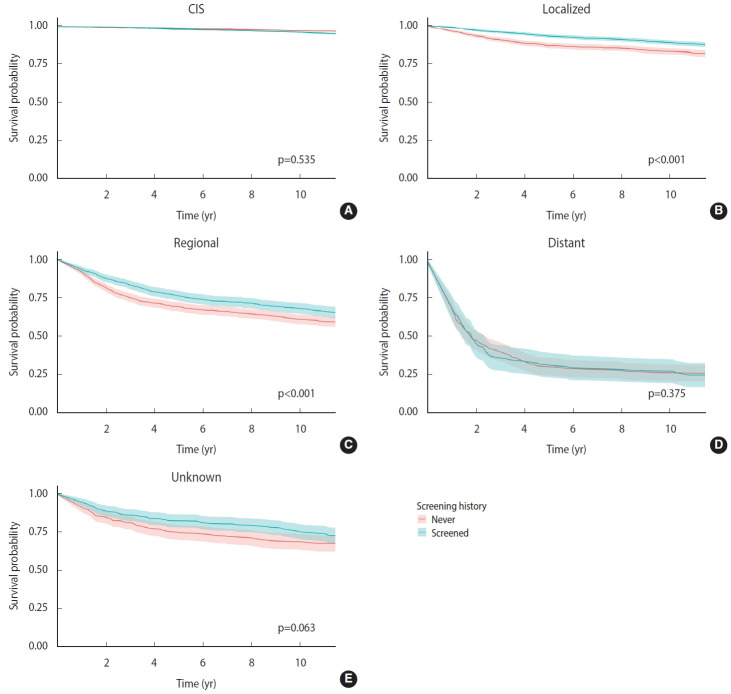
Long-term survival of the study population according to screening history by stage at diagnosis. (A) Carcinoma in situ, (B) localized, (C) regional, (D) distant, and (E) unknown.

Regarding long-term survival, 87.4% of patients were still alive at the end of the follow-up period (median, 10.5 years; interquartile range, 10.5-11.5 years) (Supplementary Material 1). Patients in the younger age group and higher socioeconomic status had higher survival than those in the other groups. Depending on the stage of cancer, the survival of cervical cancer patients varied considerably. While approximately 97% patients with early-stage disease (CIS) survived, the survival rate was lower (85.6%) in patients with localized disease and even lower (25.7%) in those with distant metastasis. Patients in the screened group had a higher survival rate (89.8%) than those in the never-screened group (84.2%). Although the survival of patients in the screened and never-screened groups was similar in the CIS (p=0.535) and distant stage (p=0.375) subgroups, significantly higher survival was observed in the screened group among patients with localized and regional-stage disease (Figure 1).

Table 2 presents the HRs for total death, cervical cancer-specific death, and non-cervical cancer death among patients. In the univariate analysis, an over 50% lower risk of cervical cancer death was observed (HR, 0.43; 95% CI, 0.38 to 0.48). After fully adjusting for socio-demographic and tumor characteristics, the risk reduction was 38% (adjusted hazard ratio [aHR], 0.62; 95% CI, 0.54 to 0.70). The aHRs for total mortality and non-cervical cancer mortality were 0.70 and 0.82, respectively. In the analysis with invasive cancer patients alone, while a very similar effect of screening for cervical cancer-specific mortality was observed (39% risk reduction), no significant difference in the risk of non-cervical cancer mortality was noted (Supplementary Material 2).

The subgroup analysis by stage at diagnosis indicated that the Pap smear screening did not reduce the risk of cervical cancer-related deaths in patients diagnosed with CIS (aHR, 1.12; 95% CI, 0.26 to 4.82) and distant-stage disease (aHR, 0.88; 95% CI, 0.67 to 1.15). However, screening significantly reduced the risk of cervical cancer death in patients with localized, regional, and unknown stages, by 59%, 35%, and 38%, respectively (Table 3). Regarding the subgroups by age at diagnosis, except for patients aged 30-39 years, a significant reduction in cervical cancer-specific mortality was observed in all age groups, and the maximum reduction was observed in women aged 50-59 (aHR, 0.54; 95% CI, 0.42 to 0.69) (Table 3).

HRs for cervical cancer-specific death according to screening frequency and time interval since the last screening are presented in Table 4. The risk of death from cervical cancer decreased with an increase in the number of screening rounds, with aHRs ranging from 0.67 (95% CI, 0.58 to 0.77) in women who underwent 1 round of screening to 0.52 (95% CI, 0.40 to 0.67) in women screened 3 times or more. A similar trend was also observed when we assessed the time interval since the last screening. Those who underwent the last cervical cancer screening within 2 years prior to cancer diagnosis had a 46% lower risk of cervical cancer death compared to those who did not (aHR, 0.54; 95% CI, 0.47 to 0.63). The risk reduction decreased as the screening interval increased and fell to only 21% when the last screening was 36-59 months prior to cancer diagnosis. In addition, there was no significant reduction in cervical cancer death among patients screened 60 months or more before the date of a cancer diagnosis.

## DISCUSSION

Our research highlighted the significant improvement in the long-term survival of screened versus never-screened CIS or invasive cervical cancer patients. The screened patients had a 38% lower risk of cervical cancer death than the never-screened. Screening significantly reduced the risk for cervical cancer-related death in all cancer stage subgroups except CIS and the distant stage. In particular, cervical cancer patients with localized and regionalized stages who underwent screening had a 59% and 35% reduction in the risk of cervical cancer death, respectively.

A population-based study that included women aged 25-65 years in Italy reported that cervical cancer patients who were never invited to or never attended a screening program had approximately double the risk of death [23]. The Finnish Cervical Cancer Screening Program indicated a 66% reduction (odds ratio, 0.34; 95% CI, 0.14 to 0.49) in the risk of cervical cancer death from screening among patients aged 25-69 years [15]. A similar effect was also indicated in the study of Vicus et al. [24], wherein women aged 30 or older received a 40-72% risk reduction from screening within 2-36 months from the diagnosis date, depending on their age group. In a Japanese study, a lower risk of cervical cancer death was also observed, with an HR of 0.30 (95% CI, 0.12 to 0.74) [13]. Although the significance of early screening is recognized in our study as well as in the previous studies, a larger risk reduction was observed in previous studies. This difference is attributed to the relatively small number of cervical cancer deaths and/or the shorter follow-up period of 5 years in the previous studies, whereas this study had a longer follow-up time.

The screening interval is one of the important aspects of screening policies, reflecting policy-makers’ attempts to maintain a balance between the benefits and harms of screening in close consideration of the resources of the health care systems. Our study found the largest risk reduction in cervical cancer death in women who received a Pap smear screening within 2 years before diagnosis, and then the effect of reducing the risk of cervical cancer death substantially decreased with a longer time interval since the last screening. No statistically significant reduction in cervical cancer death among people screened more than 5 years before a cancer diagnosis was observed. A similar effect of the time interval was also observed in previous studies [11,14,23,24]. The WHO and European Commission recommended a time interval of 3-5 years for cytology-based testing [6,7]. The Canadian Task Force on Preventive Health Care and the United States Preventive Service Task Force recommends cytology tests at 3-year intervals [4,8]. In contrast, some Asian countries such as Japan, Thailand, and Korea recommend and offer cytology tests within a 2-year interval, which is also in very good agreement with current studies [9,16]. However, to reach a conclusion on the appropriate screening interval for cervical cancer, a future study designed specifically to investigate the optimal interval of cervical cancer screening in the target population would be needed.

Regarding the screening effect by age group, we found no significant reduction in cervical cancer deaths among patients aged 30-39 years at cancer diagnosis. Similarly, no significant association was found in women aged 30-39 years in the Finnish Cervical Cancer Screening Program [15] and the population-based study by Vicus et al. [24]. In Korea, cervical cancer mortality is lower among younger women, and the rate increases gradually from the age of 35-39 [25]. Thus, cervical cancer death is minimal among women in younger age groups. Further, as an example of a cancer type characterized by relatively slow progression, women at a younger age are more prone to being diagnosed with early-stage cancer (CIS) with excellent survival, which accounted for 78.6% of cervical cancer patients aged 30-39 in our study. Lastly, as observed in our study and previous studies [16,26], the age range of 30-39 has the lowest screening participation rate, a key factor for the effectiveness of screening. This could partially explain the minimal or non-existent effect of screening in this age group. Future studies should also investigate the effect of screening both on cervical cancer incidence and mortality to generate more comprehensive evidence on screening in younger women.

Screening itself possesses some limitations including lead-time, length-time, and selection biases, which are also limitations of the current study. Regarding lead-time bias, in which the improvement in survival of screened patients is more likely associated with an earlier diagnosis rather than screening, our study had the advantage of long-term follow-up of at least 10 years for all participants. Therefore, the effect of lead-time bias on our study results was minimized. The length-time bias is related to the fact that cancer cases detected by screening are less likely to involve progression. Lastly, selection bias refers to the differences in the characteristics of the screened and never-screened populations, affecting the outcomes. To partially control these biases, we applied multilevel adjustments and then also conducted a stratification analysis to assess variation in the screening effect across subgroups. Additionally, the HRs for all-cause mortality and all-cause mortality except cervical cancer were also reported as the net benefit from screening in the cohort study using the formula [*net benefit*=*(HR^b^-HR^a^)/HR^b^ × 100*] [27], where *HR^b^* represents the HR for total mortality except cervical cancer-specific mortality, and *HR^a^* reflects the HR for cervical cancer-specific mortality. Accordingly, the net benefits of Pap smear screening were 24.4%, 27.4%, 44.6%, and 27.8% for all cancer cases, invasive cervical cancer cases only, localized-stage patients, and regional-stage patients, respectively (Table 2 and Supplementary Material 3).

Besides screening-related issues, the current study has some additional limitations. First, our study only covered screening provided through the KNCSP. The women who received opportunistic screening might be included in the never-screened group, consequently underestimating the screening effect. The screening effect might have also been underestimated, as our study could not exclude symptomatic patients, who are more likely to exhibit an advanced stage and a worse prognosis at screening. Secondly, since only the SEER summary stage is used as a staging index in cancer registry data [17], our analysis was limited to this summary staging rather than other detailed staging systems [28,29]. Additionally, our study could not adjust for treatment information, human papillomavirus infection, and other related risk factors, as we covered all invasive and CIS cervical cancer patients diagnosed in Korea in 2008 and 2009. Future studies should carefully consider these factors. However, in the context of all Korean residents enrolling in the NHIS and the universal screening program in Korea, with a lifetime screening rate of more than 70% [16], we believe that the distribution of those factors among the screened and never-screened populations is likely to be similar. Lastly, only patients diagnosed in 2008 and 2009 were recruited to ensure an appropriate observation period for screening and survival. Future studies should consider investigating patients diagnosed in later years in the cancer screening program to provide more comprehensive evidence. Despite these limitations, this study provides real-world evidence of the effect of Pap smear screening on the long-term survival of cervical cancer patients using individuallevel data from the most reliable data sources from 3 national databases [16,17], which is a direct and appropriate method to assess the efficacy of population-based screening for cervical cancer [30].

In conclusion, the current study reports the significant effects of Pap smear screening on the long-term survival of cervical cancer patients, which persisted in a subgroup analysis by cancer stage. Furthermore, patients who were screened within 2 years before the diagnosis had the best survival. In addition, women aged 50-59 years showed the largest risk reduction in cervical cancer-related mortality. Women aged 70-79 years also showed a significant risk reduction, suggesting that women in these age groups need to continue being screened.

## Figures and Tables

**Table 1.** Baseline characteristics of cervical cancer patients diagnosed from 2008 to 2009 according to their screening history

Characteristics		Total (n=14,867)	Never-screened (n=6,449)	Screened (n=8,418)	p-value
Age at diagnosis (yr)					
	30-39	2,561 (17.2)	1,695 (26.3)	866 (10.3)	<0.001
	40-49	5,726 (38.5)	2,529 (39.2)	3,197 (38.0)	
	50-59	3,131 (21.1)	1,087 (16.9)	2,044 (24.3)	
	60-69	2,083 (14.0)	617 (9.6)	1,466 (17.4)	
	70-79	1,366 (9.2)	521 (8.1)	845 (10.0)	
Socioeconomic status					
	NHIS upper 50%	5,934 (39.9)	2,265 (35.1)	3,669 (43.6)	<0.001
	NHIS lower 50%	8,090 (54.4)	3,841 (59.6)	4,249 (50.5)	
	MAP	843 (5.7)	343 (5.3)	500 (5.9)	
Cancer stage					
	CIS	8,708 (58.6)	3,439 (53.3)	5,269 (62.6)	<0.001
	Localized	3,436 (23.1)	1,472 (22.8)	1,964 (23.3)	
	Regional	1,689 (11.4)	958 (14.9)	731 (8.7)	
	Distant	389 (2.6)	256 (4.0)	133 (1.6)	
	Unknown	645 (4.3)	324 (5.0)	321 (3.8)	
Histological subtype					
	Squamous cell carcinoma	12,150 (81.7)	5,294 (82.1)	6,856 (81.4)	<0.001
	Adenocarcinoma	853 (5.7)	382 (5.9)	471 (5.6)	
	Others	1,864 (12.5)	773 (12.0)	1,091 (13.0)	
Screening frequency					
	Never	6,449 (43.4)	6,449 (100)	-	NA
	1 time	4,526 (30.4)	NA	4,526 (53.8)	
	2 times	2,168 (14.6)	NA	2,168 (25.8)	
	3 or more	1,724 (11.6)	NA	1,724 (20.5)	
Time interval since screening (mo)					
	Never	6,449 (43.4)	6,449 (100)	-	NA
	≤23	6,605 (44.4)	NA	6,605 (78.4)	
	24-35	460 (3.1)	NA	460 (5.5)	
	36-59	739 (5.7)	NA	739 (10.1)	
	≥60	403 (3.4)	NA	403 (6.0)	
Death					
	CC death	1,162 (7.8)	734 (11.4)	428 (5.1)	<0.001
	All-cause except CC	718 (4.8)	287 (4.5)	431 (5.1)	
	Alive	12,987 (87.4)	5,428 (84.2)	7,559 (89.8)	

Values are presented as number (%). NHIS, National Health Insurance Service; MAP, Medical Aid Program; CIS, carcinoma in situ; CC, cervical cancer; NA, not available.

**Table 2.** Hazard ratios for different causes of death according to screening history

Variables		Deaths (n)	Person-years	Death rate per 1,000	Crude	Model 1^1^	Model 2^2^
All-cause death							
	Never screened	1,021	62,828.8	16.3	1.00 (reference)	1.00 (reference)	1.00 (reference)
	Screened	859	85,955.7	10.0	0.62 (0.57, 0.68)	0.45 (0.41, 0.49)	0.70 (0.64, 0.77)
CC death							
	Never screened	734	62,828.8	11.7	1.00 (reference)	1.00 (reference)	1.00 (reference)
	Screened	428	85,955.7	5.0	0.43 (0.38, 0.48)	0.33 (0.29, 0.37)	0.62 (0.54, 0.70)
Non-CC death							
	Never screened	287	62,828.8	4.6	1.00 (reference)	1.00 (reference)	1.00 (reference)
	Screened	431	85,955.7	5.0	1.10 (0.95, 1.28)	0.73 (0.63, 0.85)	0.82 (0.71, 0.96)

Values are presented as hazard ratio (95% confidence interval). CC, cervical cancer.

1 Adjusted for age and socioeconomic status.

2 Adjusted for age, socioeconomic status, stage, and histological subtype.

**Table 3.** HRs for cervical cancer death stratified by age group and stage at diagnosis according to screening history

Variables			No. of CC deaths	Person-years	Death rate per 1,000	HR (95% CI)
Stage at diagnosis^1^						
	CIS					
		Never screened	3	37,222.9	0.1	1.00 (reference)
		Screened	7	56,306.3	0.1	1.12 (0.26, 4.82)
	Localized					
		Never screened	171	14,257.8	12.0	1.00 (reference)
		Screened	114	19,910.4	5.7	0.41 (0.32, 0.52)
	Regional					
		Never screened	306	7,535.2	40.6	1.00 (reference)
		Screened	166	6,233.7	26.6	0.65 (0.54, 0.79)
	Distant					
		Never screened	179	1,067.2	167.7	1.00 (reference)
		Screened	84	559.8	150.0	0.88 (0.67, 1.15)
	Unknown					
		Never screened	75	2,745.6	27.3	1.00 (reference)
		Screened	57	2,945.5	19.4	0.62 (0.44, 0.89)
Age at diagnosis (yr)^2^						
	30-39					
		Never screened	59	17,882.4	3.3	1.00 (reference)
		Screened	11	9,258.3	1.2	0.70 (0.35, 1.39)
	40-49					
		Never screened	190	25,928.8	7.3	1.00 (reference)
		Screened	81	33,778.0	2.4	0.71 (0.54, 0.92)
	50-59					
		Never screened	184	10,190.1	18.1	1.00 (reference)
		Screened	105	21,097.1	5.0	0.54 (0.42, 0.69)
	60-69					
		Never screened	118	5,414.7	21.8	1.00 (reference)
		Screened	97	14,574.5	6.7	0.62 (0.47, 0.82)
	≥70					
		Never screened	183	3,412.8	53.6	1.00 (reference)
		Screened	134	7,247.7	18.5	0.61 (0.49, 0.77)

HR, hazard ratio; CC, cervical cancer; CIS, carcinoma in situ; CI, confidence interval.

1 Adjusted for age, socioeconomic status, and histological subtype.

2 Adjusted for socioeconomic status, stage, and histological subtype.

**Table 4.** HRs for cervical cancer death according to screening frequency and time interval since the last screening

Variables		No. of CC deaths	Person-years	Death rate per 1000	Fully adjusted HR (95% CI)^1^
Screening frequency				
	Never screened	734	62,828.8	11.7	1.00 (reference)
	1 time	257	45,947.9	5.6	0.67 (0.58, 0.77)
	2 times	106	22,148.5	4.8	0.57 (0.46, 0.70)
	3 times or more	65	17,859.4	3.6	0.52 (0.40, 0.67)
Time interval since screening (mo)				
	Never screened	734	62,828.8	11.7	1.00 (reference)
	≤23	255	68,064.1	3.7	0.54 (0.47, 0.63)
	24-35	35	4,559.4	7.7	0.69 (0.49, 0.97)
	36-59	75	8,637.7	8.7	0.76 (0.60, 0.96)
	≥60	63	4,694.5	13.4	0.82 (0.64, 1.09)

HR, hazard ratio; CC, cervical cancer; CI, confidence interval.

1 Adjusted for age, socioeconomic status, stage, and histological subtype.

## SUPPLEMENTARY MATERIALS

Supplementary Material 1. Long-term survival status of cervical cancer patients

Supplementary Material 2 Hazard ratios for all-cause death, CC death, and non-CC death according to screening history

Supplementary Material 3 Hazard ratios for all-cause death, CC death, and non-CC death to screening history for invasive cancer only (N=6,159)
