# Supplementary material for: Effect of Pap smears on the long-term survival of cervical cancer patients: a nationwide population-based cohort study in Korea
Source: Epidemiol Health. 2022 Sep 7;44:e2022072. doi: 10.4178/epih.e2022072 (PMC9943631; doi:10.4178/epih.e2022072)
Supplement: Supplementary Material 1. — Long-term survival status of cervical cancer patients [file epih-44-e2022072-Supplementary-1.docx]

**Supplementary Materials**

Supplementary Material 1. Long-term survival status of cervical cancer patients

|  | **Long–term survival** | | |  | **Hazard ratios^1^** |
| --- | --- | --- | --- | --- | --- |
| **Variables** | **Alive** | **CC death** | **Non-CC death** | **P-value** | cHR (95%CI) |
|  | n=12987 (%) | n= 1162 (%) | n= 718 (%) |  |  |
| **Age at diagnosis** |  |  |  |  |  |
| 30–39 | 2467 (96.3) | 70 (2.7) | 24 (0.9) | <0.001 | 1.00 |
| 40–49 | 5362 (93.6) | 271 (4.7) | 93 (1.6) |  | 1.75 (1.4–2.20) |
| 50–59 | 2743 (87.6) | 289 (9.2) | 99 (3.2) |  | 3.54 (2.82–4.43) |
| 60–69 | 1669 (80.1) | 215 (10.3) | 199 (9.6) |  | 5.86 (4.68–7.32) |
| 70–79 | 746 (54.6) | 317 (23.2) | 303 (22.2) |  | 15.74 (12.67–19.55) |
| **Socioeconomic status** | |  |  |  |  |
| NHIS upper 50% | 5268 (88.8) | 379 (6.4) | 287 (4.8) | <0.001 | 1.00 |
| NHIS lower 50% | 7104 (87.8) | 658 (8.1) | 328 (4.1) |  | 1.10 (1.00–1.21) |
| MAP | 615 (73.0) | 125 (14.8) | 103 (12.2) |  | 2.69 (2.32–3.13) |
| **SEER Stage** |  |  |  |  |  |
| CIS | 8429 (96.8) | 10 (0.1) | 269 (3.1) | <0.001 | 0.21 (0.18–0.24) |
| Localized | 2942 (85.6) | 285 (8.3) | 209 (6.1) |  | 1.00 |
| Regional | 1061 (62.8) | 472 (27.9) | 156 (9.2) |  | 3.04 (2.70–3.42) |
| Distant | 100 (25.7) | 263 (67.6) | 26 (6.7) |  | 10.65 (9.20–12.33) |
| Unknown | 455 (70.5) | 132 (20.5) | 58 (9.0) |  | 2.27 (1.92–2.69) |
| **Histological subtype** |  |  |  |  |  |
| Squamous | 10704 (88.1) | 880 (7.2) | 566 (4.7) | <0.001 | 1.00 |
| Adenocarcinoma | 661 (77.5) | 137 (16.1) | 55 (6.4) |  | 2.04 (1.75–2.37) |
| Others | 1622 (87.0) | 145 (7.8) | 97 (5.2) |  | 1.10 (0.96–1.26) |
| **Screening history** |  |  |  |  |  |
| Never | 5428 (84.2) | 734 (11.4) | 287 (4.5) | <0.001 | 1.00 |
| Ever | 7559 (89.8) | 428 (5.1) | 431 (5.1) |  | 0.62 (0.57–0.68) |
| **Screening frequency** |  |  |  |  |  |
| Never | 5428 (84.2) | 734 (11.4) | 287 (4.5) | <0.001 | 1.00 |
| 1 time | 4034 (89.1) | 257 (5.7) | 235 (5.2) |  | 0.66 (0.60–0.74) |
| 2 times | 1945 (89.7) | 106 (4.9) | 117 (5.4) |  | 0.62 (0.54–0.72) |
| 3 or more | 1580 (91.6) | 65 (3.8) | 79 (4.6) |  | 0.50 (0.42–0.60) |
| **Time interval since screening** | |  |  |  |  |
| Never | 5428 (84.2) | 734 (11.4) | 287 (4.5) | <0.001 | 1.00 |
| ≤23 months | 6025 (91.2) | 255 (3.9) | 325 (4.9) |  | 0.53 (0.48–0.59) |
| 24–36 months | 392 (85.2) | 35 (7.6) | 33 (7.2) |  | 0.92 (0.72–1.18) |
| 36–60 months | 739 (86.8) | 75 (8.8) | 37 (4.3) |  | 0.80 (0.66–0.98) |
| ≥60 months | 403 (80.3) | 63 (12.5) | 36 (7.2) |  | 1.28 (1.05–1.58) |
| *NHIS, National Health Insurance Service; MAP, Medical Aid Program; CIS, Carcinoma in situ; CC, Cervical cancer; cHR, crude hazard ratio; 95% CI, 95% confidence interval.*  *^1^ Hazard ratio for all-cause deaths* | | | | | |
