# Supplementary material for: Effect of Pap smears on the long-term survival of cervical cancer patients: a nationwide population-based cohort study in Korea
Source: Epidemiol Health. 2022 Sep 7;44:e2022072. doi: 10.4178/epih.e2022072 (PMC9943631; doi:10.4178/epih.e2022072)
Supplement: Supplementary Material 2 — Hazard ratios for all-cause death, CC death, and non-CC death according to screening history [file epih-44-e2022072-Supplementary-2.docx]

**Supplementary Materials**

Supplementary Material 2. Hazard ratios for all-cause death, CC death, and non-CC death according to screening history

|  | **All-cause death** | **CC death** | **Non–CC death** |
| --- | --- | --- | --- |
|  | HR (95% CI) | HR (95% CI) | HR (95% CI) |
| **Stage at diagnosis^1^** |  |  |  |
| **CIS** |  |  |  |
| Never screened | 1.00 | 1.00 | 1.00 |
| Screened | 0.77 (0.59–1.00) | 1.12 (0.26–4.82) | 0.75 (0.57–0.99) |
| **Localized** |  |  |  |
| Never screened | 1.00 | 1.00 | 1.00 |
| Screened | 0.53 (0.44–0.63) | 0.41 (0.32–0.52) | 0.74 (0.56–0.98) |
| **Regional** |  |  |  |
| Never screened | 1.00 | 1.00 | 1.00 |
| Screened | 0.72 (0.61–0.85) | 0.65 (0.54–0.79) | 0.90 (0.66–1.24) |
| **Distant** |  |  |  |
| Never screened | 1.00 | 1.00 | 1.00 |
| Screened | 0.97 (0.76–1.25) | 0.88 (0.67–1.15) | 2.62 (1.17–5.89) |
| **Unknown** |  |  |  |
| Never screened | 1.00 | 1.00 | 1.00 |
| Screened | 0.65 (0.48–0.87) | 0.62 (0.44–0.89) | 0.71 (0.42–1.20) |
| **Age at diagnosis (years) ^2^** |  |  |  |
| **30–39** |  |  |  |
| Never screened | 1.00 | 1.00 | 1.00 |
| Screened | 0.79 (0.47–1.34) | 0.70 (0.35–1.39) | 1.19 (0.51–2.79) |
| **40–49** |  |  |  |
| Never screened | 1.00 | 1.00 | 1.00 |
| Screened | 0.80 (0.64–1) | 0.71 (0.54–0.92) | 1.09 (0.72–1.65) |
| **50–59** |  |  |  |
| Never screened | 1.00 | 1.00 | 1.00 |
| Screened | 0.65 (0.53–0.8) | 0.54 (0.42–0.69) | 1.16 (0.75–1.78) |
| **60–69** |  |  |  |
| Never screened | 1.00 | 1.00 | 1.00 |
| Screened | 0.66 (0.54–0.81) | 0.62 (0.47–0.82) | 0.70 (0.51–0.94) |
| **≥70** |  |  |  |
| Never screened | 1.00 | 1.00 | 1.00 |
| Screened | 0.65 (0.55–0.76) | 0.61 (0.49–0.77) | 0.70 (0.55–0.89) |
| *CC, Cervical cancer; CIS, Carcinoma in situ; HR, hazard ratio; 95% CI, 95% confidence interval.*  *^1^ Adjusted for age, socioeconomic status, and histological subtype.*  **^2^** *Adjusted for socioeconomic status, stage, and histological subtype.* | | | |
