# Supplementary material for: Effect of Pap smears on the long-term survival of cervical cancer patients: a nationwide population-based cohort study in Korea
Source: Epidemiol Health. 2022 Sep 7;44:e2022072. doi: 10.4178/epih.e2022072 (PMC9943631; doi:10.4178/epih.e2022072)
Supplement: Supplementary Material 3 — Hazard ratios for all-cause death, CC death, and non-CC death to screening history for invasive cancer only (N=6,159) [file epih-44-e2022072-Supplementary-3.docx]

**Supplementary Materials**

Supplementary Material 3. Hazard ratios for all-cause death, CC death, and non-CC death to screening history for invasive cancer only (N=6,159)

|  | **All-cause death** | **CC death** | **Non–CC death** |
| --- | --- | --- | --- |
|  | HR (95% CI) | HR (95% CI) | HR (95% CI) |
| **Overall^1^** |  |  |  |
| Never screened | 1.00 | 1.00 | 1.00 |
| Screened | 0.68 (0.61–0.75) | 0.61 (0.54–0.69) | 0.84 (0.70–1.02) |
| **Age at diagnosis (years)^2^** |  |  |  |
| **30–39** |  |  |  |
| Never screened | 1.00 | 1.00 | 1.00 |
| Screened | 0.72 (0.39–1.35) | 0.67 (0.33–1.37) | 1.23 (0.32–4.80) |
| **40–49** |  |  |  |
| Never screened | 1.00 | 1.00 | 1.00 |
| Screened | 0.79 (0.62–1.00) | 0.71 (0.55–0.93) | 1.25 (0.71–2.18) |
| **50–59** |  |  |  |
| Never screened | 1.00 | 1.00 | 1.00 |
| Screened | 0.62 (0.5–0.77) | 0.53 (0.41–0.68) | 1.19 (0.72–1.97) |
| **60–69** |  |  |  |
| Never screened | 1.00 | 1.00 | 1.00 |
| Screened | 0.65 (0.52–0.81) | 0.62 (0.47–0.82) | 0.69 (0.47–1.00) |
| **≥70** |  |  |  |
| Never screened | 1.00 | 1.00 | 1.00 |
| Screened | 0.66 (0.55–0.79) | 0.61 (0.49–0.77) | 0.73 (0.56–0.97) |
| *CC, Cervical cancer; CIS, Carcinoma in situ; HR, hazard ratio; 95% CI, 95% confidence interval.*  **^1^** *Adjusted for age, socioeconomic status, stage, and histological subtype.*  **^2^** *Adjusted for socioeconomic status, stage, and histological subtype* | | | |
